# Lack of Reduction of Left Ventricular Mass in Treated Hypertension: The Strong Heart Study

**DOI:** 10.1161/JAHA.113.000144

**Published:** 2013-06-21

**Authors:** Giovanni de Simone, Richard B. Devereux, Raffaele Izzo, Daniela Girfoglio, Elisa T. Lee, Barbara V. Howard, Mary J. Roman

**Affiliations:** 1 Department of Translational Medical Sciences, Federico II University, Napoli, Italy (G.S., R.I., D.G.); 2 Department of Medicine, Weill Cornell Medical College, New York, NY (G.S., R.B.D., M.J.R.); 3 Center for American Indian Health Research, University of Oklahoma, Oklahoma City, OK (E.T.L.); 4 Medstar Research Institute, Washington, DC (B.V.H.)

**Keywords:** antihypertensive therapy, blood pressure, obesity, proteinuria, ventricular hypertrophy

## Abstract

**Background:**

Hypertensive left ventricular mass (LVM) is expected to decrease during antihypertensive therapy, based on results of clinical trials.

**Methods and Results:**

We assessed 4‐year change of echocardiographic LVM in 851 hypertensive free‐living participants of the Strong Heart Study (57% women, 81% treated). Variations of 5% or more of the initial systolic blood pressure (SBP) and LVM were categorized for analysis. At baseline, 23% of men and 36% of women exhibited LV hypertrophy (LVH,* P*<0.0001). At the follow‐up, 3% of men and 10% of women had regression of LVH (*P*<0.0001 between genders); 14% of men and 15% of women, free of baseline LVH, developed LVH. There was an increase in LVM over time, more in men than in women (*P*<0.001). Participants whose LVM did not decrease had similar baseline SBP and diastolic BP, but higher body mass index (BMI), waist/hip ratio, heart rate (all *P*<0.008), and urinary albumin/creatinine excretion (*P*<0.001) than those whose LVM decreased. After adjusting for field center, initial LVM index, target BP, and kinship degree, lack of decrease in LVM was predicted by higher baseline BMI and urinary albumin/creatinine excretion, independently of classes of antihypertensive medications, and significant effects of older age, male gender, and percentage increase in BP over time. Similar findings were obtained in the subpopulation (n=526) with normal BP at follow‐up.

**Conclusions:**

In a free‐living population, higher BMI is associated with less reduction of hypertensive LVH; lack of reduction of LVM is independent of BP control and of types of antihypertensive treatment, but is associated with renal damage.

## Introduction

Clinical trials almost invariably indicate that reduction of hypertensive left ventricular mass (LVM) is an achievable goal during antihypertensive management^1–5^ and that this reduction results in a decreased incidence of cardiovascular (CV) events,^6–7^ independently of reduction in blood pressure (BP) and other factors that decrease CV risk. However, the translation of these findings into clinical practice is made difficult by a number of critical issues, including selection of patients, definition of hypertensive LV hypertrophy (LVH), and relation to BP control.^8^ There are also issues related to the standardized trial environment, which is not automatically reproducible in real life clinical practice; thus observational studies are sometimes needed to assess the applicability of randomized study findings to the general population.^9–10^

The reduction of LVM reported in randomized clinical trials is variable in relation to selected populations, type of medication, duration of trial, and type of design, but can be estimated to be 8% to 14% over 2 to 48 months of treatment.^11^ Whether such an effective reduction of LVM can be achieved in usual clinical contexts or in unselected free‐living populations is often assumed, but remains to be proven. Accordingly, this analysis has been designed to compare changes in LVM over a 4‐year follow‐up in the unselected cohort of treated hypertensive participants in the Strong Heart Study (SHS), and to identify predictors of modifications in LVM.

## Methods

### Population

The SHS is a longitudinal population‐based survey of cardiovascular risk factors and disease in American Indians from 13 communities in Arizona, Oklahoma, and South and North Dakota.^12^ The fourth SHS examination, conducted between 2001 and 2003, enrolled 3658 individuals who were members of large 3‐generation families (the Strong Heart Family Study), of which 520 were members of the original SHS cohort.^13–14^ Among them, 1133 hypertensive participants without valve regurgitation greater than mild and without valve stenosis of any degree were identified, 862 (76%) of whom underwent a repeated echocardiogram after 4 years at the time of the fifth SHS exam. For the purpose of this analysis, participants with triglycerides >750 mg/dL were excluded (n=11), consistent with our previous reports.^15^ Thus, the analyzed population sample included 851 participants, of whom 488 (57%) were women.

### Procedures

Clinical examinations, including a personal interview, physical exam, and morning blood sample collection after a 12‐hour fast were performed at local community settings and Indian Health Service clinics by the study staff. Detailed descriptions of the study design and methods of the SHS have been previously reported.^12–14^

Brachial systolic and diastolic BP (SBP and DBP) were measured 3 consecutive times on seated participants using appropriately sized cuffs. The mean of the last 2 of these measurements was used to record BP levels. Diabetes mellitus (DM) was defined by fasting glucose ≥126 mg/dL or use of insulin or oral hypoglycemic therapy.

### Echocardiographic Measures

Echocardiograms were performed by expert sonographers, according to standardized methods, and reviewed offline by 2 independent readers,^16^ following American Society of Echocardiography recommendations.^17^ The LVM was calculated by a necropsy‐validated formula^18^ and was normalized for height in meter to the power of 2.7, an allometric signal that linearizes the curvilinear relation between LVM and height across a wide age range.^19^ LVH was defined using a nonsex‐specific population‐specific partition values, which maximizes the population risk attributable to LVH (47.24 g/m^2.7^).^20^

Variations of at least 5% of the initial values of both SBP or LVM index (LVMi, in g/m^2.7^) were categorized for analysis.

### Statistical Analysis

Data were analyzed using SPSS 20.0 (IBM). Indicator variables were included in all multivariable analyses for the 3 field centers. Exploratory statistics were run to find the potential confounders to be used in multivariable analyses, using chi‐square distribution for categories (with Monte Carlo method for computation of exact 2‐tailed *P* value, when appropriate) analysis of variance and least square linear regression. Full‐factorial 2‐way analysis of variance for repeated measures was used to verify the time course of variables potentially influencing variations of LVM and to explore possible sex‐related differences. Baseline demographic and metabolic characteristics were compared in male and female participants with or without reduction of LVM, using 2‐way analysis of covariance, adjusted for baseline LVMi and the percent changes in SBP.

The impact of family relatedness was considered in multivariable analyses, as previously reported^21^ by using standard kinship coefficients (0.25 for parent/offspring, 0.25 for full siblings, 0.125 for half siblings, 0 for no consanguinity). Binary, multivariable logistic regression was used to identify initial characteristics of participants who did not change or increased LVMi over time, using a hierarchical model in 3 steps. In the first step, critical adjusting variables were entered (age, sex, family relatedness, baseline blood pressure and change in blood pressure). In a second step, a backward stepwise procedure was run including all variables that, at baseline, differed between participants with or without reduction of LVMi, to identify a phenotype with high probability of preserving or increasing LVM. In the third step, classes of antihypertensive medications were forced into the model to verify whether antihypertensive treatment could modify the phenotype associated with lack of reduction of LVM. In multivariable logistic analysis modeling, kinship coefficient was first entered together with the other critical adjusting variables.

## Results

The population sample (Table 1) was characterized by a slight predominance of women, who were younger than men (45±15 versus 54±14 years, *P*<0.0001), with a high prevalence of overweight and obesity, and a high prevalence of diabetes. Eighty‐one percent of the hypertensive participants were on antihypertensive medications.

**Table 1.** Demographic Characteristics of the SHS Hypertensive Participants Who Repeated Echocardiogram 4 Years Apart

N	851
Age, y	49±14
BMI, kg/m^2^	31±6
Sex (% women)	57
Body size, %	
Normal weight	17
Overweight	35
Obesity	48
Central fat distribution, %	76
Diabetes, %	43
Untreated hypertension, %	19
Current smokers, %	30

SHS indicates Strong Heart Study; BMI, body mass index.

### Characteristics of the Population Sample

Compared with the baseline (fourth) exam, SBP decreased in 343 (40%) participants, increased in 270 (32%) and remained unchanged in 238 (28%). On average, SBP decreased significantly only in men (Figure), whereas DBP decreased significantly in both men and women (all *P*<0.0001). Table 2 includes the initial distribution of antihypertensive medications in the 3 subgroups of participants. The subgroup with increase in follow‐up BP were more likely to take ACE‐inhibitors, β‐blockers, and Ca^2+^‐channel blockers than the other subgroups, whereas no difference was found for diuretics, angiotensin II‐receptor blockers, α‐blockers and other medications.

**Table 2.** Distribution of Antihypertensive Medications at the Time of the 4th SHS Exam in Participants Exhibiting Reduced, Stable, or Increased Blood Pressure 4 Years Later (5th Exam)

Medications	BP↓ >5% of Baseline (n=343)	BP≈ (Within 5% Variation) (n=238)	BP↑ >5% of Baseline (n=270)	*P* Value
ACE‐inhibitors	41%	38%	53%	≤0.001
ARB	3.8%	5.5%	5.2%	≤0.58
β‐Blockers	8.7%	6.3%	15.2%	≤0.002
CCB	13.4%	13.4%	21.1%	≤0.02
Diuretics	19.5%	16.4%	21.9%	≤0.30
α‐Blockers	3.2%	1.7%	1.5%	≤0.28
Others	3.2%	1.3%	3.3%	≤0.27

SHS indicates Strong Heart Study; BP, blood pressure; ACE, angiotensin‐converting enzyme; ARB, angiotensin receptor blockers; CCB, Ca^2+^ channel blockers.

**Figure 1.**
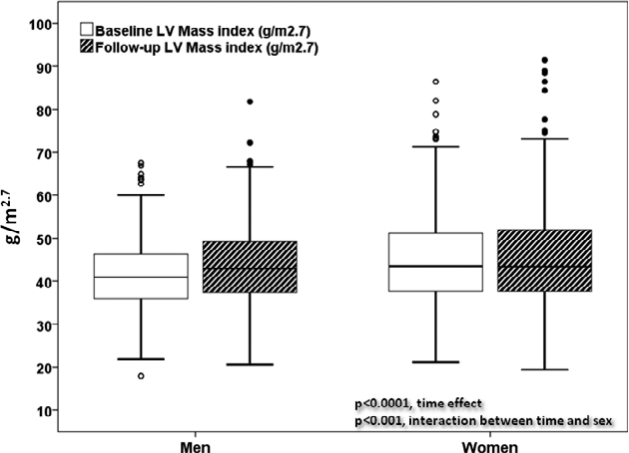
Side‐to‐side box plots of left ventricular (LV) mass index at baseline (white boxes) and after 4‐year follow‐up (dashed boxes) in treated hypertensive women and men, participants of the Strong Heart Study.

Body mass index (BMI) was similar in women and men and was unchanged after 4 years (Table 3), but in men there was a tendency to increase. Waist/hip ratio increased especially in women. Triglycerides decreased in both men and women. Glomerular filtration rates decreased similarly in men and women and urinary albumin/creatinine ratio slightly increased. While DBP was reduced in both genders, SBP decreased significantly more in men than in women. No other significant changes or interactions could be detected.

**Table 3.** Initial and Follow‐Up CV Profile in Female and Male Participants in the Present Study

	Men (n=363), 54±14 Years		Women (n=488), 45±15 Years	
	Baseline	4‐Year Follow‐Up	Baseline	4‐Year Follow‐Up
Heart rate, bpm	69.1±11.7	68.8±11.4	68.8±10.8	68.2±10.4
BMI, kg/m^2^	33.6±6.7	34.1±7.8	34.3±7.1	34.5±7.3
Waist/hip ratio**	0.98±0.07	0.99±0.06	0.91±0.06	0.94±0.06
Systolic BP**, mm Hg	137±17	132±18	135±18	134±21
Diastolic BP*, mm Hg	87±12	80±13	79±13	72±13
Fasting glucose, mmol/L	6.94±3.00	7.32±3.44	7.55±3.61	7.60±3.72
HDL‐cholesterol, mmol/L	1.27±0.42	1.21±0.37	1.38±0.39	1.33±0.38
Triglycerides*, mmol/L	2.16±1.27	1.92±1.13	2.08±1.02	1.85±1.06
GFR*, mL/min per 1.73 m^2^	100±28	93±33	88±29	78±32
Urinary albumin/creatinine*	10.2 (5.5 to 35.5)	11.0 (5.2 to 61.9)	12.5 (6.8 to 41.8)	14.0 (6.8 to 36.3)

CV indicates cardiovascular; BMI, body mass index; BP, blood pressure; HDL, high‐density lipoprotein; GFR, glomerular filtration rate.

* 0.02<*P*<0.0001, time effect.

* 0.003<*P*<0.001 interaction between time and sex.

### Change of LVMi Over Time

At the time of the baseline exam LVH was found in 262 (31%) participants and in 309 (36%) at the follow‐up exam. At baseline, 23% of men and 36% of women exhibited LVH (*P*<0.0001 between genders). At the time of the follow‐up exam only 3% of men and 10% of women had clear‐cut LVH regression (*P*<0.0001 between genders). In contrast, 14% of men and 15% of women who did not have LVH at the baseline developed LVH during the 4 years of follow‐up.

Average LVMi increased from the fourth (43.8±9.9 g/m^2.7^) to the fifth exam (44.9±10.5 g/m^2.7^, *P*=0.0001). This increase was due to greater changes in men than in women (*P*<0.001 between genders). Baseline LVMi was significantly greater in participants decreasing than in those increasing or maintaining their LVM during follow‐up (44.3±9.8 and 41.4±8.4 g/m^2.7^ in men; 47.0±11.7 and 44.5±9.9 g/m^2.7^ in women, respectively, *P*<0.002). Percent change of LVMi was weakly related to percent change of SBP (r=0.18) and follow‐up SBP (r=0.19) and DBP (r=0.14; all *P*<0.01).

We performed a sex‐specific comparison between participants decreasing or not decreasing (or increasing) their LVMi during follow‐up, adjusting for changes in SBP, for baseline values of LVMi and for degree of family relatedness (Table 4). Participants who did not decrease their LVM were slightly, but not significantly, older than those with reduction of LVM. In addition, they had similar SBP and DBP, substantially higher BMI, waist/hip ratio, and heart rate (all *P*<0.004), and significantly higher urinary albumin/creatinine ratio (*P*<0.001). No differences could be found in fasting glucose and lipid profile. The differences between average values of BMI and waist/hip ratio in participants with or without reduction of LVM were marginally greater in men than in women, but no other gender‐differences were observed.

**Table 4.** Initial Characteristics in Male and Female SHS Participants With or Without Reduction of LVMi During Follow‐Up

	Men (n=363), 54±14 Years		Women (n=488), 45±15 Years		*P* Value < for Change in LVM	*P* Value < for Interaction Sex‐Change
	Reduction of LVM (n=67)	No Reduction or Increase of LVM (n=296)	Reduction of LVM (n=127)	No Reduction or Increase of LVM (n=361)		
Age, y	44±14	45±15	52±14	54±14	0.22	0.60
Prevalence of diabetes, %	33	36	46	47	NS	NS
Systolic BP, mm Hg	139±19	137±16	136±19	134±18	0.62	0.63
Diastolic BP, mm Hg	89±13	86±12	80±12	79±13	0.37	0.23
Heart rate, bpm	66±11	70±12	67±10	69±11	0.004	0.46
BMI, kg/m^2^	31.1±5.5	34.2±6.8	33.7±6.8	34.4±7.2	0.0001	0.04
Waist/hip ratio	0.96±0.06	0.99±0.07	0.90±0.06	0.91±0.06	0.001	0.05
Fasting glucose, mmol/L	6.55±2.22	7.05±3.16	7.27±3.44	7.60±3.61	0.15	0.50
HDL‐cholesterol, mmol/L	1.32±0.46	1.26±0.41	1.40±0.36	1.38±0.40	0.30	0.40
Triglycerides, mmol/L	2.15±1.23	2.16±1.28	2.10±1.02	2.08±1.02	0.87	0.89
GFR, mL/min per 1.73 m^2^	101±27	100±28	91±30	87±28	0.33	0.83
Urinary albumin/creatinine	7.7 (5.2 to 21.5)	10.8 (5.6 to 39.1)	11.9 (6.6 to 30.3)	13.0 (7.0 to 49.4)	0.003	0.77

Except for age and prevalence of diabetes, comparisons are adjusted for changes in systolic BP, baseline values of LVMi and degree of family relatedness. SHS indicates Strong Heart Study; LV indicates left ventricle; NS, not significant; BP, blood pressure; LVMi, left ventricular mass index; HDL, high‐density lipoprotein; GFR, glomerular filtration rate.

Lack of decrease in LVMi was not related to any class of medications used at the baseline (data not shown). In multivariable, multistep logistic regression, adjusting for field center, age, sex, degree of relatedness, initial LVMi, follow‐up BP, and change of SBP as percent of baseline values (Table 5), lack of decrease in LVMi was associated with initially higher BMI and urinary albumin/creatinine excretion independently of significant effects of older age, male gender, and change in BP over time, and without additional contribution of initial waist/hip ratio. Forcing all classes of antihypertensive medications into the model did not substantially modify the coefficients displayed in Table 5 (data not shown).

**Table 5.** Predictors of Lack of Reduction of LVMi in Treated Hypertensive Subjects

	B	*P* Value	OR	95% CI for OR (Lower to Upper)
Age, y	0.02	<0.007	1.02	1.01 to 1.04
Female sex (n/y)	−0.57	<0.003	0.56	0.38 to 0.83
Degree of family relatedness*	0.86	<0.45	2.36	0.25 to 22.0
Baseline LV mass index, g/m^2.7^	−0.06	<0.0001	0.94	0.92 to 0.96
Baseline BMI, kg/m^2^	0.08	<0.0001	1.08	1.05 to 1.12
Baseline systolic BP, mm Hg	0.02	<0.03	1.02	1.002 to 1.03
Baseline heart rate, bpm	0.01	<0.09	1.02	1.00 to 1.03
Baseline urinary albumin/creatinine, log_10_	0.49	<0.001	1.63	1.21 to 2.19
Change in systolic BP, % of baseline	0.04	<0.0001	1.04	1.02 to 1.06
Follow‐up hypertension (n/y)	−0.49	<0.08	0.61	0.36 to 1.06
Constant	−2.29	<0.07	—	—

LVMi indicates left ventricle mass index; LV, left ventricle; CI, confidence interval; OR, odds ratio; BMI, body mass index; BP, blood pressure.

* Kinship coefficients: 0.25 for parent/offspring, 0.25 for full siblings, 0.125 for half siblings, and 0 for no consanguinity.

As a confirmation, we analyzed the subpopulation (n=526, 301 women) that exhibited target BP at the follow‐up (ie, SBP <140 and DBP <90: 122±11/71±11 mm Hg). Among the 151 participants who had baseline LVH (29%), 129 (85%) remained with LVH at the follow‐up, compared to the 22 (15%) who exhibited regression of LVH (*P*<0.0001). The same logistic model displayed in Table 5 was therefore performed in this subpopulation, resulting in the same pattern of risk as shown in Table 5, with high baseline BMI (OR=1.08/kg×m^−2^; 95% CI=1.04 to 1.12, *P*<0.0001) and log_10_ urinary albumin/creatinine (OR=1.78; 95% CI=1.23 to 2.56, *P*<0.002) as the markers of risk of not reducing initial LVH despite good control of BP (both *P*<0.0001), without additional effect of change of SBP (*P*=0.247).

## Discussion

The results of the present analysis, performed in a free‐living sample of treated hypertensive adults with high prevalence of obesity and diabetes, suggest that antihypertensive management may not effectively decrease LVM in usual clinical care programs. The lack of effect is associated with older age, initial central obesity, and kidney damage, but not with the type of antihypertensive therapy. This analysis strongly suggests that (1) in real‐life context, persistent obesity inhibits the attempt to reduce LVM; (2) the lack of reduction of LVM in this setting is at least in part independent of BP control and types of initial antihypertensive medications, and is associated with renal damage; and (3) results of clinical trials on regression of LVH cannot be automatically applied to unselected free‐living populations receiving standard programs of primary cardiovascular prevention.

The effect of obesity on BP control and reduction of LVM has been increasingly examined. Obesity and its associated metabolic abnormalities have been shown to substantially reduce the chance of effective BP control, despite more aggressive antihypertensive management.^21–22^ In the CampaniaSalute Network, we have shown that the presence of multiple metabolic risk factors (including obesity, lipid abnormalities and impaired fasting glucose), substantially reduces the chance to achieve optimal BP control, despite more aggressive management.^23^ Similar results were recently reported by the French Nutrition and Health Survey,^24^ which highlighted that the difficulty in the antihypertensive management could not be imputed to inadequate treatment. This negative effect on BP is also translated into target organ damage. In the Losartan Intervention for Endpoint Reduction in Hypertension (LIFE) study, clusters of metabolic risk factors, including obesity, were associated with less reduction of electrocardiogram‐LVH in both diabetic and nondiabetic groups.^25^ Similar findings were produced in 875 patients recruited in the LIFE echo substudy.^25–26^ One of the reasons in the lack of reduction of LVM in obesity might be in the myocardial composition of obese subjects. The nonmuscular component of myocardium in obesity is likely to be large, as a number of studies suggest,^27^ being formed of adipocytes and preadipocytes in addition to possibly large population of fibroblasts, all cell components that do not respond (or respond much less than cardiomyocytes) to modification of loading conditions.

The partial independence of modification of LVM from loading conditions is sustained by the evidence that the lack of reduction of LVMi in the SHS is substantially independent of BP control. This independence was already evident in the multivariable logistic analysis, but was eventually demonstrated by analyzing all participants with effective BP control at the end of follow‐up, which confirmed the results found in the entire population sample. Thus the association with obesity cannot be attributable to lack of BP control or adherence to medications. In contrast, lack of reduction of LVM is clearly associated with renal damage. These findings are made even more relevant because the rate of BP control at the time of follow‐up was excellent (62%), as compared to baseline (n=360 or 42%, *P*<0.0001), and greater than generally reported in the literature.^28^ The discrepancy between BP control and persistence of initial values of LVMi is consistent with a number of previous findings, suggesting that the paradigm of BP‐LVH as cause effect relation should be revised to recognize the role of potentially interfering parameters.^29^ Increased LVM has been shown to precede development of arterial hypertension in a number of clinical and epidemiological studies,^30–33^ a finding that supports a reverse‐causation speculation^29,34^ and helps explain why therapeutic intervention may control BP (the effect), and much less LVM (one of the potential determinants under this scenario).

Unfortunately a more extensive analysis of the potential effect of therapy could not be done because only the association with the initial treatment could be analyzed leaving a lack of information on variation of treatment during the follow‐up. It is interesting that all major classes of medications tended to be used more in those participants who did not exhibit reduction of LVM, suggesting that already at the beginning of the study more aggressive therapy was indicated. Also, day‐to‐day or even week‐to‐week variability of BP and LVM could be important, but would unlikely be known in a real‐world context.

Our results are obtained in normal community‐based settings, without the typical randomized controlled trial (RCT) restraints. This is the greatest difference from the RCT environment, which is usually confined to patients with some specific characteristics.

Finally, the generalizability of findings from RCTs has been questioned by many researchers, and the need for translational research and postmarketing studies has been increasingly emphasized.^9–10,9–36^ Our findings raise speculation about the applicability of results of clinical trials to clinical practice. Cuspidi et al^8^ highlighted that the results of trials on regression of LVH are not necessarily automatically applicable in clinical practice, due to a number of factors, including the variability of the definition of hypertensive LVH and the lack of information on rates of regression of LVH or reduction of LVM, 2 terms that are not necessarily equivalent.^37^ In addition, by design, clinical trials of regression of hypertensive LVH have enrolled patients with BP levels that could be substantially reduced (eg, by 22/13 mm Hg in Prospective Randomized Enalapril Study Evaluating Regression of Ventricular Enlargement (PRESEVE)^38^ and by 25/15 mm Hg in the LIFE echo substudy,^1^ both of which showed substantial LVH regression).

The selection made by many clinical trials, designed to assess efficacy of therapy on reduction of LVM or regression of LVH, is rigorous and often the study‐cohorts do not reflect the variety of circumstances and conditions presenting with hypertensive patients in clinical practice. This is particularly evident for obesity, because, in a number of echocardiographic studies, there was a lower prevalence of obesity in the study cohort than in free‐living hypertensives, due to well‐known technical problems in performing high‐quality echocardiograms that yield accurate, reproducible measurements.^39^ The ability to obtain readable echocardiograms in obese patients is increasing over time,^40^ but is still below that of nonobese members of the general population,^41^ and may be lower in unselected clinical outpatient hypertensive populations than in participants in trials of LVH regression. The SHS cohort provides a unique opportunity because of the very high rate of readable echocardiograms, despite the very high prevalence of obesity.^42^

Our findings need to be considered with caution, because the specific characteristics of the SHS population (ethnicity, prevalence of obesity and diabetes) preclude generalization. However, preliminary findings from the CampaniaSalute network, a large registry of whites from southern Italy,^43^ suggest that the relations between hypertensive LVM and BMI might be very similar to what has been reported in the participants of the SHS.^44^

## Conclusions

This analysis demonstrates that, in a free‐living sample of hypertensive participants from a population‐based study with high prevalence of obesity, hypertensive LVH is commonly sustained or may develop despite effective antihypertensive therapy, contrary to expectations from results of randomized controlled trials. Persistence or development of LVH in treated hypertensive adults appears to be independent of blood pressure control and is associated with persistent obesity and renal damage.
